# Correction: Antonuccio et al. The Nutraceutical N-Palmitoylethanolamide (PEA) Reveals Widespread Molecular Effects Unmasking New Therapeutic Targets in Murine Varicocele. *Nutrients* 2021, *13*, 734

**DOI:** 10.3390/nu15071662

**Published:** 2023-03-29

**Authors:** Pietro Antonuccio, Herbert Ryan Marini, Antonio Micali, Carmelo Romeo, Roberta Granese, Annalisa Retto, Antonia Martino, Salvatore Benvenga, Salvatore Cuzzocrea, Daniela Impellizzeri, Rosanna Di Paola, Roberta Fusco, Raimondo Maximilian Cervellione, Letteria Minutoli

**Affiliations:** 1 Department of Human Pathology of Adult and Childhood, University of Messina, 98125 Messina, Italy; 2 Department of Clinical and Experimental Medicine, University of Messina, 98125 Messina, Italy; 3 Department of Biomedical and Dental Sciences and Morphofunctional Imaging, University of Messina, 98125 Messina, Italy; 4 Department of Chemical, Biological, Pharmaceutical and Environmental Sciences, University of Messina, 98166 Messina, Italy; 5 Department of Paediatric Urology, Royal Manchester Children’s Hospital, Oxford Road, Manchester M13 9WL, UK

## Error in Figure

In the original publication [1], there was a mistake in Figures 2 and 3 as published. The authors presented a factual error in the figures, copying and pasting the same information twice in two different places. In the original version, Figures 2I and 3D displayed an identical histological image. In the correction, both have been replaced. The corrected Figure 2 and Figure 3 appear below. The authors apologize for any inconvenience caused and state that the scientific conclusions are unaffected. This correction was approved by the Academic Editor. The original publication has also been updated.

## Figures and Tables

**Figure 2:**
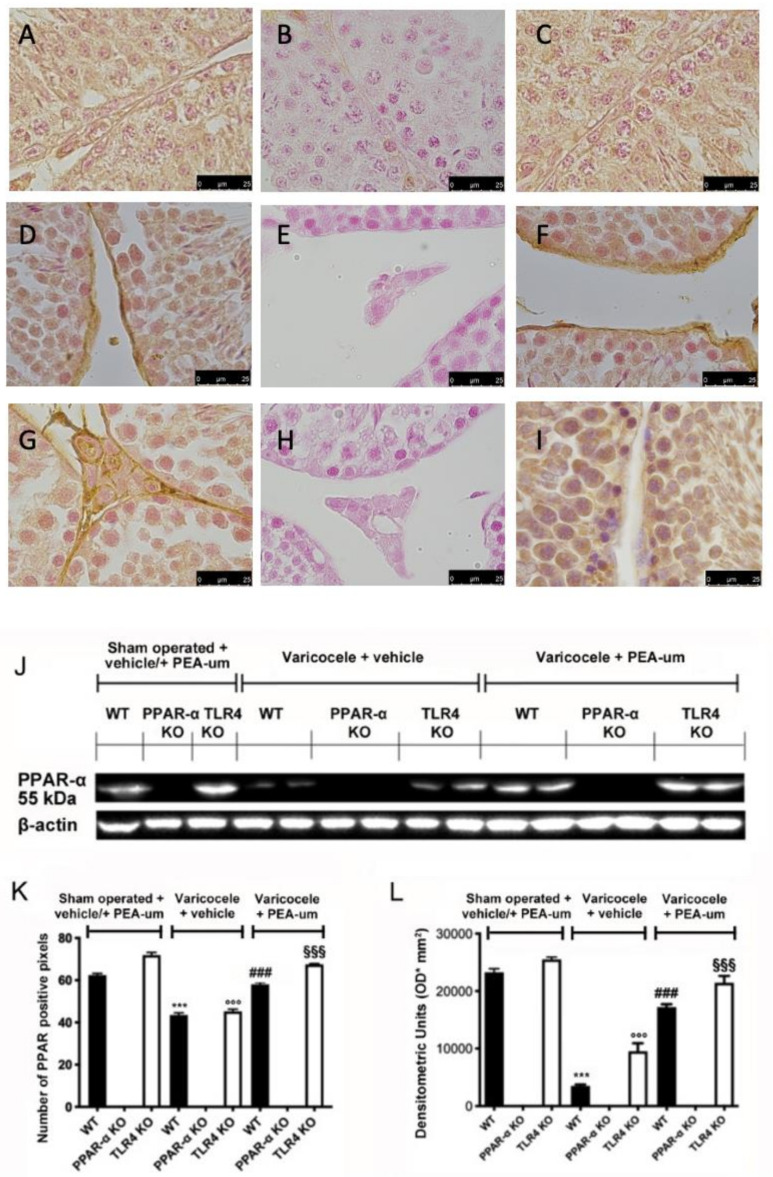
Effects of the absence of PPAR-α and TLR4 on PPAR-α expression. Immunohistochemical evaluation of PPAR-α expression. (**A**): Sham operated WT; (**B**): Sham operated PPAR-α KO; (**C**): Sham operated TLR4 KO; (**D**): Vehicle varicocele WT; (**E**): Vehicle varicocele PPAR-α KO; (**F**): Vehicle varicocele TLR4 KO; (**G**): PEA-um varicocele WT; (**H**): PEA-um varicocele PPAR-α KO; (**I**): PEA-um varicocele TLR4 KO; (**J**): Densitometric analysis; (**K**): Western blot analysis of PPAR-α expression; (**L**): densitometric analysis. Scale bar 100×. *** *p* < 0.001 vs. sham WT, ^###^ *p* < 0.001 vs. vehicle WT; ^°°°^ *p* < 0.001 vs. sham TLR4, ^§§§^ *p* < 0.001 vs. vehicle TLR4.

**Figure 3:**
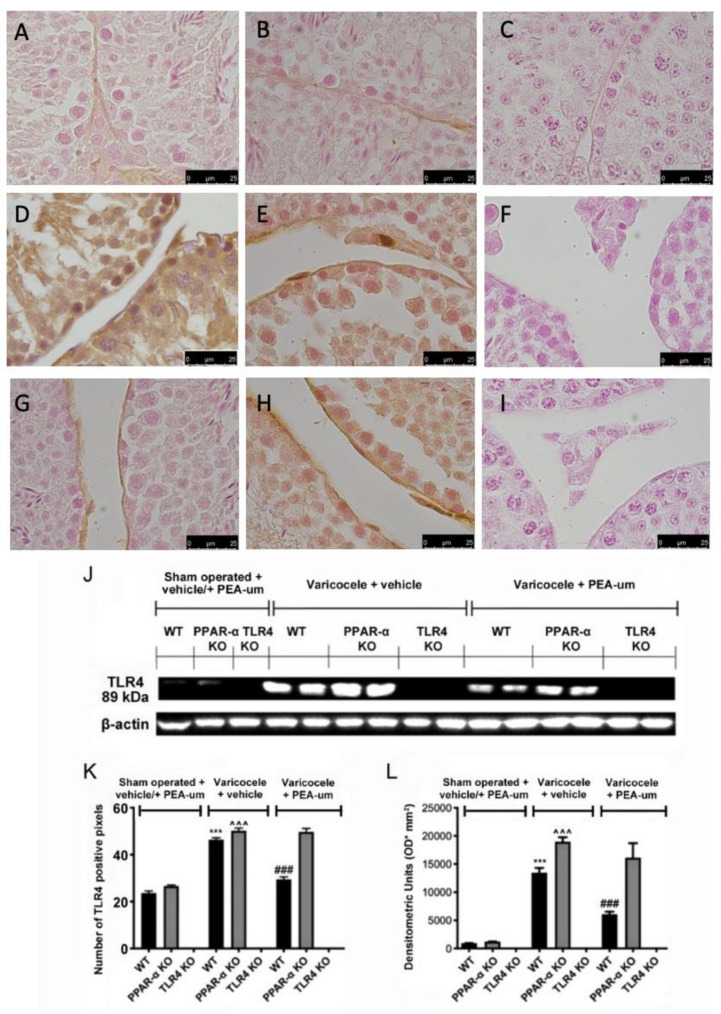
Effects of the absence of PPAR-α and TLR4 on TLR4 expression. Immunohistochemical evaluation of TLR4 expression. (**A**): Sham operated WT; (**B**): Sham operated PPAR-α KO; (**C**): Sham operated TLR4 KO; (**D**): Vehicle varicocele WT; (**E**): Vehicle varicocele PPAR-α KO; (**F**): Vehicle varicocele TLR4 KO; (**G**): PEA-um varicocele WT; (**H**): PEA-um varicocele PPAR-α KO; (**I**): PEA-um varicocele TLR4 KO; (**J**): Densitometric analysis; (**K**): Western blot analysis of TLR4 expression; (**L**): densitometric analysis. Scale bar 100×. *** *p* < 0.001 vs. sham WT, ^###^ *p* < 0.001 vs. vehicle WT ˆˆˆ *p* < 0.001 vs. sham PPAR-α.
